# Spatial Heterogeneity of Soil Chemical Properties in a Subtropical Karst Forest, Southwest China

**DOI:** 10.1155/2014/473651

**Published:** 2014-02-12

**Authors:** Zhonghua Zhang, Baoqing Hu, Gang Hu

**Affiliations:** ^1^Key Laboratory of Beibu Gulf Environment Change and Resources Utilization of Ministry of Education, Guangxi Teachers Education University, Nanning 530001, China; ^2^School of Chemistry and Life Science, Guangxi Teachers Education University, Nanning 530001, China

## Abstract

This study evaluates the spatial heterogeneity of the soil chemical properties of surface soils across a 1 ha old-growth subtropical karst forest in southwest China.

## 1. Introduction

Soils are formed by physical, chemical, and biological processes that act upon the geological parent material and the continuous interaction of these processes with the biotic, climatic, and topographic components of the environment [1, 2]. These components often cause the heterogeneity in soil properties at the spatial and temporal scales [3–5]. Previous studies investigated the relationships between the spatial heterogeneity of soil properties and the environmental factors in different landscape types [1, 6–9]. Many studies demonstrated that topographic factors, such as slope percentage, aspect, elevation, and microrelief, play important roles in determining the spatial variation in soil properties at different scales [10, 11]. However, the spatial variability of soil properties and the complex relationship between soil and topographic factors in different ecosystems or landscape types remain barely understood [12, 13]. Soil properties, such as key factors, affect plant distribution, community dynamics, and even the structure and function of ecosystems [14, 15]. Thus, analyses on the spatial heterogeneity of soil properties in different plant communities can contribute well to the understanding of the structure and function of soil. In addition, these analyses can explore the relationship between soil properties and plant diversity in an ecosystem.

A karst ecosystem is defined as an ecosystem that is restrained by a karst environment [16], especially by karst geological settings [17]. Carbonate rock is the material basement of a karst ecosystem, and its matter migration and energy transfer have their own particularities, such as soluble rock, calcium-rich, and double-layer hydrogeological structures, which are different from other ecosystems within the same climate zone [18]. The karst ecosystem in China mainly covers the southwestern regions, such as Guizhou, Yunnan, and Guangxi. A karst topography is a geological formation shaped by the dissolution of a layer or layers of soluble bedrocks, which are usually carbonate rocks, such as limestone or dolomite [16]. The existence of special landforms, such as the peak cluster, peak forest, low-lying land, and the funnel, cause significant changes in the topographic factors, such as elevation, slope, and aspect [19]. Moreover, various micro-reliefs, such as stone facing, stone trench, and swallet, formed by abundant rock outcrops dramatically influence the small-scale habitat heterogeneity [5]. Therefore, topographic factors possibly have an important influence on the spatial variability of soil properties in the karst region. However, studies on the spatial variability of soil properties in karst hill slopes and its relationship with topographic factors remain unavailable.

A karst mixed evergreen-deciduous broadleaved forest was chosen in the Maolan National Nature Reserve (MNNR) in southwest China because of its rich biological diversity and diverse topography. In the present study, the spatial heterogeneity of pH and soil nutrients in surface soils (0 cm to 10 cm deep) across 1 ha (100 m × 100 m) of old-growth subtropical karst forest was evaluated. The spatial variability and the spatial patterns of the soil chemical properties at the plot scale were characterized using a combination of classical and geostatistical methods. The objectives of this study are as follows: (1) to determine the spatial variability characteristics of the soil chemical properties of a subtropical karst forest and (2) to examine the correlations between the spatial distribution of the soil chemical properties and the local topographic variables.

## 2. Materials and Methods

### 2.1. Site Description

**Figure 1:**
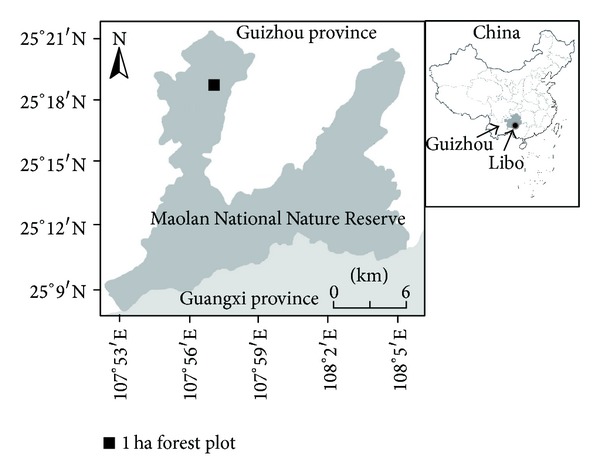
Location of the 1 ha forest plot in the Maolan National Natural Reserve in Guizhou province, southwest China.

The study area is located in the MNNR (25°09′20′′ to 25°20′50′′N, 107°52′10′′ to 108°05′40′′E), Libo County, Guizhou province, in southwest China (Figure 1). The reserve is approximately 200,000 ha in size and has an elevation in the range from 430 m to 1,078.6 m, with an average of 800 m. A subtropical monsoon climate dominates the area with a mean annual rainfall of 1,320.5 mm. The mean temperature ranges from 8.3°C in January to 26.4°C in July, with an annual mean of 15.3°C. The annual evaporation is 1,343.6 mm, and the annual mean relative humidity is 83%. Carbonate rocks are usually exposed on the surface, and the soils are thin and discontinuous in the study area. The shallow, black limestone soil is rich in organic matter and nutrients (N, P, K, and Ca).

### 2.2. Soil Sampling and Measurements

The 1 ha (100 m × 100 m) plot was established in a typical old-growth mixed evergreen-deciduous broadleaved forest in the MNNR in the summer of 2008. The plot is located at a steep southeast-facing hill slope (mean slope of ca. 45°) from the valley bottom to the hilltop at an elevation in the range from 835 m to 912 m. Rock outcrops occur on almost the entire plot (ca. 85% of the ground surface). *Castanopsis carlesii *var.* spinulosa*, *Cyclobalanopsis myrsinifolia*, *Platycarya longipes*, *Distylium myricoides*, *Rhododendron latoucheae*, *Osmanthus fragrans*, *Engelhardtia roxburghiana*, *Sloanea sinensis*, and *Carpinus pubescens* dominate the vegetation at the plot. Using the DQL-1 forest compass (Harbin Optical Instrument Factory, China), the plot was divided into 100 contiguous 10 m × 10 m quadrats. Soil samples at depths in the range from 0 cm to 10 cm at three locations were chosen randomly within each 10 m × 10 m quadrat. The three soil samples in each quadrat were then mixed, and therefore a total of 100 soil samples were collected from the plot. Moreover, the elevation, slope degree and aspect, and the percentage of rock bareness were recorded. Elevation was measured using a portable GPS (GPSMAP 60CSx, Garmin Ltd., Taiwan, China). The slope degree and aspect were measured using a DQL-1 Forest Compass. The percentage of rock bareness (basement rock was not covered by soil and was exposed on the ground surface) within each 10 m × 10 m quadrant was visually estimated.

All soil samples were transported to the laboratory for chemical analysis. Each soil sample was air-dried and passed through a 2 mm sieve to separate fine earth and coarse soil fractions. All subsequent analyses were performed on the fine fractions. Ten chemical properties were analyzed according to the methods described in Bao [20]. Soil pH was measured in a 1 : 2.5 soil-to-water suspension. Soil organic matter (OM) was measured using the K_2_Cr_2_O_7_-capacitance method, total nitrogen (TN) was measured using the micro-Kjeldahl method, total phosphorus (TP) was measured using NaOH fusion and Mo-Sb colorimetric procedures, total potassium (TK) was measured using NaOH fusion and flame photometry, and total calcium (TCa) and total magnesium (TMg) were measured using atomic absorption spectrometry. The available nitrogen (AN) in the soil was determined using the diffusion-absorption method. Available phosphorus (AP) was extracted using NaHCO_3_ solution and its content was determined using the Mo-Sb colorimetric method. Available potassium (AK) was extracted with neutral ammonium acetate and was measured using flame photometry.

### 2.3. Data Analysis

Both the classical statistics and geostatistics were used to analyze the spatial features of the measured variables. Conventional statistics was used to indicate the degree of overall variation, while geostatistics was used to examine whether or not a variable is spatially structured.

The normality of all data sets was tested prior to the conventional statistical and geostatistical analyses. Data were log or square-root transformed when the normality test failed. Conventional statistics, that is, mean (median for skewed data), standard deviation (SD), and coefficient of variation (CV), was performed to indicate the overall variability of each analyzed item. Spearman correlation coefficients were used to determine the relationships between soil chemical properties and topography factors, such as elevation, slope degree, slope aspect, and rock bareness rate. All the analyses above were performed using the statistical software package SAS (version 9, SAS Institute Inc, Cary, NC, USA).

The spatial patterns of the measured variables were analyzed using GS^+^ software (version 5.3.2, Gamma Design Software, Plainwell, MI, USA) for semivariogram computation and kriging. This analysis produces variograms that reveal random and structured aspects of the spatial dependence in a dataset of multiple samples collected at increasing distances from each other (the lag interval). The variogram plots of the semivariance statistic *γ*(*h*) for a range of distance intervals *h* can be expressed as follows:

(1) $$\begin{array}{c} \gamma \left(h\right)=\frac{1}{2N\left(h\right)}\overset{N(h)}{\underset{i=1}{\sum }}{\left[Z(x_{i})-Z(x_{i}+h)\right]}^{2}, \end{array}$$

where *γ*(*h*) is the semivariance, *N*(*h*) is the number of observation pairs separated by a distance *h*, *Z*(*χ*_*i*_) is the value of the variable of interest at location *χ*_*i*_, and *Z*(*χ*_*i*_ + *h*) is the value of the variable of interest at a location at distance *h* from *χ*_*i*_.

In this study, three geostatistical models, namely, spherical, exponential, and linear models, were used to evaluate the resulting semivariograms. Three semivariogram parameters, namely, the nugget (*C*_0_), the range, and the ratio of structure variance (*C*) and sill variance (*C*_0_ + *C*) (SH%, hereafter), were derived and used in the analysis. *C*_0_ reflects either the variability at scales finer than the data resolution or the random error. The range indicates the spatial autocorrelation distance between data pairs. SH% represents the proportion of variance caused by spatial dependence. Semivariogram with a high SH% indicates a strong spatial structure [21, 22]. Maps of the soil properties were produced with GS^+^ software, following the ordinary block kriging with a block size of 2 m × 2 m. The transformed data (logarithm or square root) prior to the semivariogram analysis were converted back to their original units prior to kriging [23].

## 3. Results and Discussion

### 3.1. Descriptive Statistics

The data were analyzed using classical statistical methods to understand the characteristics of the general soil properties prior to the investigation on the spatial structure (Table 1). The minimum, maximum, difference between median and average, SD, and CV can describe the variability of a soil property. The results of the Kolmogorov-Smirnov test (*K*-*S* test) (*P* = 0.05 probability level) indicate that the soil properties data were distributed normally, except for TN, TCa, and AK, and that these variables were subjected to log transformation (Table 1).

The mean surface soil pH was slightly alkaline. The CV for the soil properties was in the range of 5%–65%. AP was the most variable among all the soil properties (CV = 63.9%). TP, TCa, and AK were also highly variable. Nielsen and Bouma [24] suggested the following three distinct classes of variability for soil properties based on CV values: 0%–15% indicates little variability; 10%–100% indicates moderate variability; and > 100% indicates high variability. In the current study, the soil variability data in Table 1 show that all the CVs, except for soil pH, were between 10% and 65%. These data indicate that soil chemical properties in the study site were moderately variable at the local scale. Many studies reported that soil pH is the least variable soil property [25]. The current study also showed that soil pH in the karst forest has low variability (CV = 5.5%). Similar results were found in other karst forest types [9, 26]. This moderate variability of soil properties may be attributed to the soil processes of eluviation in karst habitats [26].

### 3.2. Spatial Variability of Soil Chemical Properties

**Figure 2:**
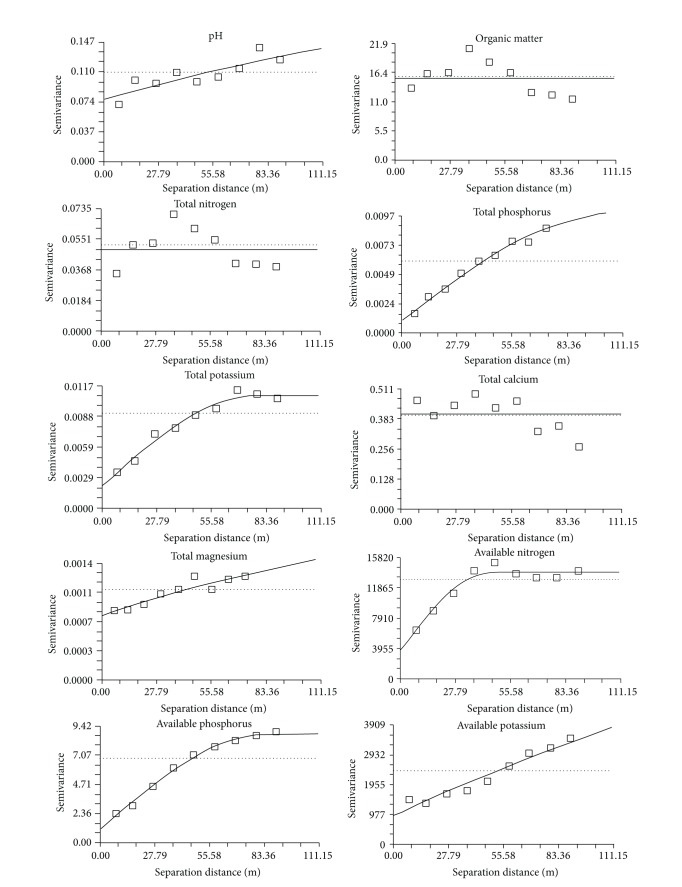
Semivariograms of the soil chemical properties on the top forest soil (0 cm to 10 cm).

The semivariogram model and some of the geostatistical parameters of soil chemical properties are shown in Table 2. A spherical model provided a significant fit (based on largest *r* ^2^ value) to the semivariogram of TP, TK, AN, AP, and AK (Table 2, Figure 2). An exponential model was selected as the best-fit model for the pH and TMg based on the regression analysis (*r* ^2^). A linear model provided the best fit to the variogram of OM, TN, and TCa. In geostatistics, mathematical models are fitted to variograms, and different models reveal the nature of the spatial pattern. For example, spherical models indicate distinct patches of large (or small) concentrations in a matrix of less (or greater) concentrations [13]. The observation that several models provide the best fit in our data indicates different spatial patterns among the set of soil properties.

The range in a variogram is the distance at which the variogram reaches the sill. The range of the semivariogram represents the average distance through which the variable semivariance reaches its peak value. The variability beyond this range does not depend on the separation distance, and the variables are no longer spatially related. The range may be viewed as the zone of influence of a variable or the transition from a state of spatial correlation to a state of absence correlation [27]. Moreover, the range is a direct measurement of the scale of spatially correlated variation. The scale of the correlated spatial variation increases with increasing range. A small, effective range implies a distribution pattern composed of small patches. In this study, the range of spatial dependence for soil chemical properties in the 0 cm to 10 cm depth (Table 2) was as small as 45.6 m for AN and 50 m to 90 m for a host of soil chemical properties. The effective ranges for pH, TMg, and AK were greater than 200 m, indicating a large-patched distribution pattern.

The *C*_0_ values show a positive nugget effect, which may be explained by the sampling error, short range variability, and random and inherent variability [28]. The difference in the degrees of structured spatial variation is indicated by the variations in the nugget effect and in the sill. The structural variance, expressed as a percentage of the total variance, allows the direct comparison of the relative strength of spatial dependence [29]. The soil variables have the following three distinct classes of spatial dependence: a ratio <25% indicates weak spatial dependence; a 25%–75% ratio indicates moderate spatial dependence; and a ratio >75% indicates strong dependence [30]. In the current study, the proportion of total variance that was spatially dependent (defined as *C*_0_/*C*_0_ + *C*) varied from <25% for TP, TK, AP, and AK, indicating that these variables have a strong spatial autocorrelation and suggesting that the parent material, terrain, and the climate are the main causes that determine strong spatial autocorrelation. The ratios of pH, TMg, and AN were between 25% and 75%. The variables had moderate spatial autocorrelations, which may be controlled by the intrinsic variations in soil characteristics (texture, mineralogy, and soil forming processes) and the effects of natural vegetation on soil [31]. These results show the different degrees of spatial variability of soil properties in karst forest soils. Different soil chemical properties occupied varied spatial autocorrelation ranges, suggesting that the spatial heterogeneity of the soil chemical properties is possibly a function of the spatial scale.

### 3.3. Spatial Distribution of the Soil Chemical Properties

**Figure 3:**
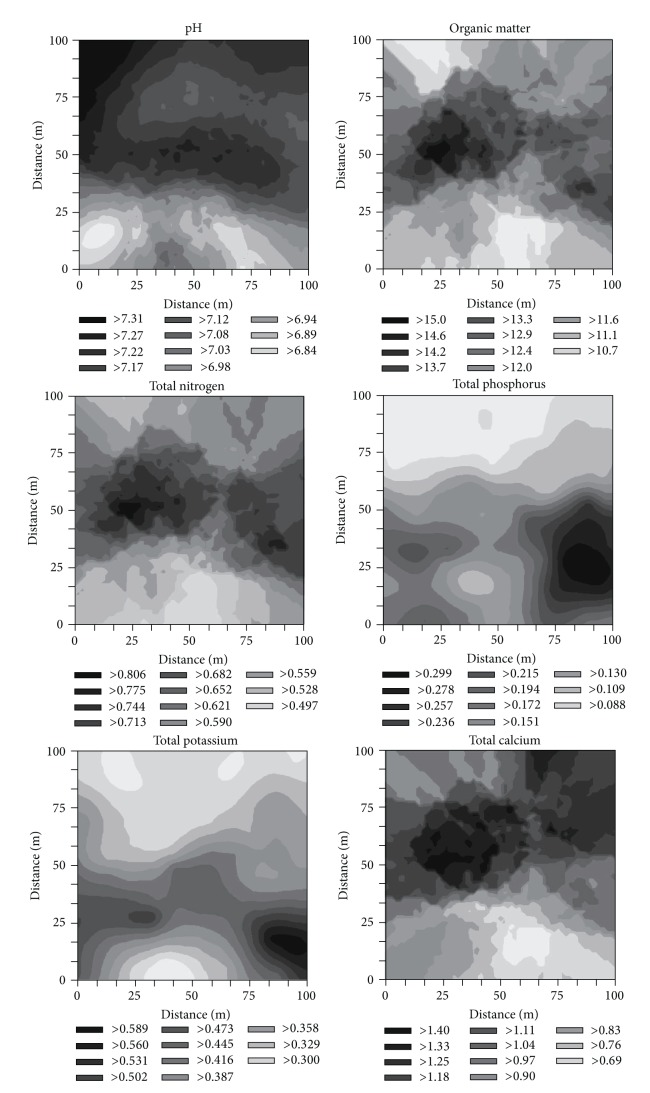

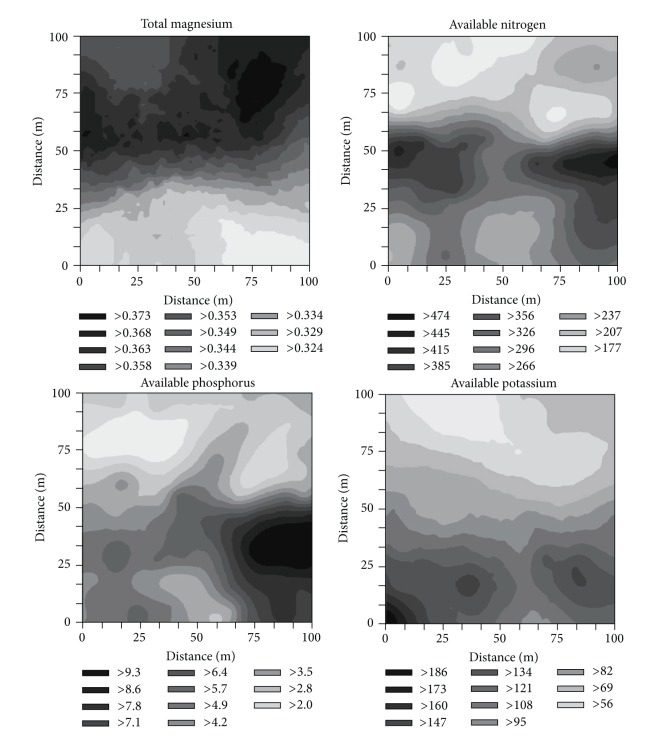
Distribution of the soil chemical properties of the entire study plot using kriging interpolation.

Using the fitted models and the corresponding parameters, a block kriging with a block size of 2 m × 2 m was performed to obtain interpolated values for all variables throughout the plot (Figure 3). Mapping the variations in the concentration of each chemical property across the study area revealed several spatially explicit patterns. For example, pH, TCa, and TMg contained large patches of higher concentrations in the middle and upper parts of the study plot, whereas patches of higher TP, TK, AN, AP, and AK concentrations were prevalent in the lower portion of the plot. OM and TN had similar spatial distributions, with higher concentrations prominent in the center of the study area. In the heterogeneous karst landscapes, the topography changed with the functions of soil nutrient, temperature, and moisture [32, 33]. In the study site, the soil properties were probably determined by the topographic factors, such as elevation, slope, and more rock outcrops.

### 3.4. Correlations between the Soil Chemical Properties and the Topographic Factors

Table 3 shows the correlations between soil properties and topographic factors in the karst forest plot. Topographic variability showed several significant correlations with different soil chemical properties (Table 3). Soil pH, TCa, and TMg showed significant and positive correlations with elevation, slope, and rock bareness rate. On the other hand, TP, TK, and AK were negatively correlated with the elevation and slope. OM and TN were positively correlated with the slope and rock bareness rate. Topography is an important factor that controls both hydrological and soil processes at the landscape scale because it affects the moisture, as well as the accumulation and export of nutrients [10, 11, 34, 35]. Some studies in the Guangxi karst regions showed a significant topographic influence on soil nutrients [36]. In the current study, the distribution of soil chemical properties in the forest plot was closely related to the topographic factors, of which the maximum values of pH, TCa, and TMg were found in the middle and upper parts of the slope of the plot and had significant positive correlation with elevation, slope, and the rock bareness rate. The middle and upper slopes present the high rock bareness rate and thin soil layer, and, thus, the weathering and eluviations of the rock contributed to the increases in soil pH, Ca, and Mg [37]. Previous studies showed that the pH value of soil is significantly correlated with the rock bareness rate [38] and that high soil pH tends to increase with increasing Ca and Mg contents [39], which is in accordance with the analytical results of the current study. The positive correlation of OM and TN with the slope and rock bareness rate should be interpreted in such a way that the soil in the rock outcrop areas often clusters in the stone trench or swallet, which are favorable for accumulating the organic matter. Zhang et al. [40] proposed that sites with high rock bareness rate and sharp slope often contain high soil nutrient, as also revealed in the results of the current study. TP, TK, and AK have an extremely significant negative correlation with the elevation and slope. The sites with low altitude and slight slope often have a low rock bareness rate and a thick soil layer, allowing them to prepare for the gradual accumulation of TP, TK, and AK. Thus, it can be concluded that pH, OM, TN, TCa, and TMg increase, while TP, TK, AN, and AK decrease with increasing elevation and the rock bareness rate. Therefore, the current study demonstrated that topographic factors, such as elevation, slope, and rock bareness rate, play important roles in determining the spatial distribution and the variability of soil chemical properties in the subtropical karst forest in southwest China.

However, topography, biology, and climate have synergistic effects on the spatial variability of soil nutrients [31]. The formation and evolution of soil fertility in forest ecosystems are significantly influenced by the biological functions of the ecosystems. The formation and sustainability of soil nutrients are based on the exuberant biological accumulation, and, in turn, the spatial variability of soil nutrients plays a part in the growth and spatial distribution of plants. Therefore, this study provided a basis for future investigations on soil-plant relationships and the ecological restoration of the degraded ecosystem in the karst region of southwest China.

## 4. Conclusions

In the current study, geostatistical methods were used to investigate the spatial heterogeneity of soil chemical properties under an old-growth subtropical karst forest in southwest China. The results of the classic statistical analysis indicate that the soil chemical properties in the study site are moderately variable at the local scale. Best-fit models to individual variograms include spherical, exponential, and linear models, indicating the different spatial patterns among the set of soil properties. A geostatistical analysis revealed spatial dependence at a scale from 10 m to 100 m for most of the soil properties. The nugget ratios of TP, TK, AP, and AK showed strong spatial autocorrelations (with the nugget/sill ratio < 0.25), indicating that the parent material, topography, and the climate are the main causes of the spatial correlations. The ratios of pH, TMg, and AN were between 25% and 75% and had moderate spatial autocorrelations, which may be controlled using the intrinsic variations in soil characteristics and the effects of natural vegetation on soil. The soil chemical properties had significantly high correlations with topography, indicating that the topographic factors, especially elevation, slope, and rock bareness rate, mainly affected the spatial distributions and variability of the soil chemical properties of a subtropical karst forest in MNNR, southwest China.

## Figures and Tables

**Table 1:** Descriptive statistics for soil chemical properties.

Soil properties	Maximum	Minimum	Mean	SD	CV (%)	*K*-*S* test (*P* = 5%)
pH	7.88	6.02	7.06	0.39	5.5	0.259
OM (%)	28.69	3.85	12.00	4.25	35.4	0.060
TN (%)	1.537	0.218	0.622	0.23	37.6	0.027
TP (%)	0.354	0.047	0.157	0.08	49.0	0.061
TK (%)	0.703	0.224	0.389	0.09	24.2	0.242
TCa (%)	3.564	0.397	1.060	0.63	59.5	0.003
TMg (%)	0.499	0.252	0.347	0.04	11.8	0.156
AN (mg/kg)	653.2	54.3	283.4	41.40	14.6	0.267
AP (mg/kg)	10.211	0.791	3.432	2.19	63.9	0.211
AK (mg/kg)	284.23	16.41	96.97	49.17	50.7	0.023

**Table 2:** Fitted model types and parameters for the semivariograms of soil chemical properties.

Soil properties	Model	Nugget (*C*_0_)	Sill (*C*_0_ + *C*)	Rang	*C*_0_/*C*_0_ + *C*	*R* ^2^
pH	Exponential	0.0760	0.2380	310.9	0.319	0.743
OM (%)	Linear	15.3355	15.3355	89.53	1.000	0.227
TN (%)	Linear	0.0495	0.0495	89.53	1.000	0.057
TP (%)	Spherical	0.0011	0.0095	121.6	0.116	0.987
TK (%)	Spherical	0.0021	0.0108	78.4	0.194	0.979
TCa (%)	Linear	0.4022	0.4022	89.53	1.000	0.538
TMg (%)	Exponential	0.0008	0.0022	206.0	0.364	0.871
AN (mg/kg)	Spherical	3610	13860	45.6	0.260	0.949
AP (mg/kg)	Spherical	1.07	8.789	84.5	0.122	0.994
AK (mg/kg)	Spherical	950	5010	213.4	0.189	0.954

**Table 3:** Correlation between the soil chemical properties and topographic factors (elevation, slope, aspect, and rock-bareness rate).

Soil properties	Elevation	Slope	Aspect	Rock-bareness rate
pH	0.227*	0.345**	−0.155	0.403**
OM (%)	0.121	0.229*	−0.106	0.343**
TN (%)	0.072	0.244*	−0.110	0.432**
TP (%)	−0.600**	−0.370**	−0.083	0.139
TK (%)	−0.558**	−0.525**	−0.180	−0.102
TCa (%)	0.223*	0.251*	−0.093	0.216*
TMg (%)	0.275**	0.230*	−0.078	0.252*
AN (mg/kg)	−0.422**	−0.129	−0.042	0.305**
AP (mg/kg)	0.042	0.138	−0.073	0.315**
AK (mg/kg)	−0.622**	−0.385**	0.109	−0.155

*P < 0.05; **P < 0.01.
